# Lactic acid bacteria in Swedish honey bees during outbreaks of American foulbrood

**DOI:** 10.1002/ece3.10964

**Published:** 2024-02-13

**Authors:** Anna Nilsson, Paul D'Alvise, Meghan O. Milbrath, Eva Forsgren

**Affiliations:** ^1^ Department of Ecology Swedish University of Agricultural Sciences Uppsala Sweden; ^2^ Institute for Clinical Microbiology and Hygiene University Hospital Tübingen Tubingen Germany

**Keywords:** American foulbrood, *Apis mellifera*, lactic acid bacteria, *Paenibacillus larvae*

## Abstract

The honey bee microbiota is involved in several important functions, and alterations in the composition could have a severe effect on honey bee health. Among the bacteria identified in the honey bee microbiome are a group of non‐pathogenic honey bee‐specific lactic acid bacteria (hbs‐LAB) that have been shown to inhibit the growth of bacterial pathogens such as *Paenibacillus larvae*, the causative agent of American foulbrood (AFB). While *P. larvae* only causes disease in larvae and not in adult honey bees, there are reports of the pathogen causing changes in the microbiota composition of the adults. The aim of this study was to investigate how AFB in the colony affect the hbs‐LAB composition in adult honey bees. Adult bees were collected from colonies with and without AFB during three outbreaks of AFB in Sweden. The hbs‐LAB was analyzed using qPCR to detect and quantify the number of ten hbs‐LAB (five *Lactobacilli*, two *Apilactobacilli*, one *Bombilactobacilli*, and two *Bifidobacterium*). The hbs‐LAB composition was compared between AFB outbreaks and depending on the AFB status of the honeybee colony at the time of sampling. The data analyses revealed differences in the abundance of individual hbs‐LAB between outbreaks and an overall difference in bacterial community composition depending on AFB status. Also, a higher hbs‐LAB diversity was observed in samples that were *P. larvae* culture positive.

## Graphical abstract

To investigate if the bacterial disease American foulbrood (AFB) affects lactic acid bacteria (LAB) in honeybees, adult bees were collected from colonies with and without AFB during outbreaks in Sweden. We found differences in the abundance of the individual LAB between the different outbreaks.

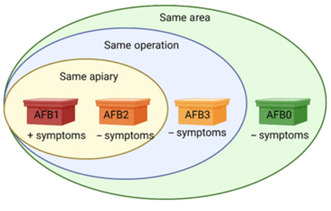

## 1 INTRODUCTION

The demand for pollinators has increased worldwide as more entomophilous flowering crops are cultivated (Aizen & Harder, 2009). At the same time, wild pollinators are in decline and there are reports of recurring high losses of managed honey bee colonies (Goulson et al., 2015; Koh et al., 2016; Neumann & Carreck, 2010; Steinhauer et al., 2018). This mismatch in supply and demand for pollination services has led to an increased interest in honey bees and has drawn attention to various factors that can affect bee health, such as honey bee‐associated microorganisms (Aizen & Harder, 2009; Gallai et al., 2009; Genersch, 2010a).

The majority of the bacterial species associated with honey bees are non‐pathogenic (Corby‐Harris et al., 2014; Cox‐Foster et al., 2007; Engel et al., 2016) and may be of great importance to honey bee health. The honey bee microbiota is involved in several important functions such as food digestion, immune system activation, and protection against pathogens, and compositional variations of the bacterial community can affect these functions (Engel et al., 2012; Khan et al., 2020; Raymann & Moran, 2018; Vásquez et al., 2012). Among the non‐pathogenic bacteria identified and isolated from honey bees are a group of functionally similar lactic acid bacteria (LAB). This group of honey bee‐specific LAB (hbs‐LAB) includes species belonging to lactobacilli, apilactobacilli, bombilactobacilli, and bifidobacteria and are in combination or individually suggested to have health‐promoting effects on honey bees (Olofsson et al., 2014, 2016; Olofsson & Vásquez, 2008). As part of the honey bee microbiota, hbs‐LAB may affect the health of honey bees in several ways. In addition to their ability to directly inhibit bacterial growth by secreted antimicrobial compounds, these bacteria can outcompete pathogens and activate the immune defense in honey bees (Engel et al., 2016; Evans & Lopez, 2004; Forsgren et al., 2010; Janashia & Alaux, 2016; Killer et al., 2014; Pachla et al., 2018; Pătruică & Mot, 2012; Truong et al., 2023; Zendo et al., 2020).

One of the most important honey bee pathogens is the spore‐forming bacterium *Paenibacillus larvae*. This bacterium causes the honey bee brood disease American foulbrood (AFB), lethal not only to individual larvae but also to entire colonies. *P. larvae* causes disease in young honey bee larvae that become infected by consuming food contaminated with infectious bacterial endospores. The spores germinate and multiply in the midgut of the larvae and then invade and putrefy the larval tissue (Genersch, 2010b). Even though *P. larvae* does not cause obvious disease in adult honey bees (Crailsheim & Riessberger‐Gallé, 2001), changes in the microbiome of adult honey bees have been reported after infection with *P. larvae* (Erban, Ledvinka, Kamler, Nesvorna, et al., 2017).

Hbs‐LAB have been shown to have an inhibitory effect on *P. larvae* both in vitro and when fed to honey bee larvae in vivo (Forsgren et al., 2010; Pachla et al., 2018; Truong et al., 2023). This inhibitory effect may suggest the possibility of using hbs‐LAB as probiotics to prevent diseases such as AFB (Pachla et al., 2018; Truong et al., 2023). However, the honey bee colony is a complex super‐organism, and effects at the individual bee level do not automatically translate to effects on the colony level (Lamei et al., 2020; Stephan et al., 2019). Previous field experiments by Lamei et al. (2020) found that feed supplements containing hbs‐LAB at the colony level had no effect on hbs‐LAB composition and abundance in the honey crop of sampled workers, but that there was higher hbs‐LAB diversity in colonies with a history of AFB compared to colonies with no history of AFB. The aim of the current study was to build on these results with a broader investigation of the composition and diversity of hbs‐LAB in samples of honey bees collected from colonies with AFB and from colonies with various AFB statuses in the surrounding area during AFB outbreaks in Sweden.

## 2 MATERIALS AND METHODS

### 2.1 Samples

**FIGURE 1:**
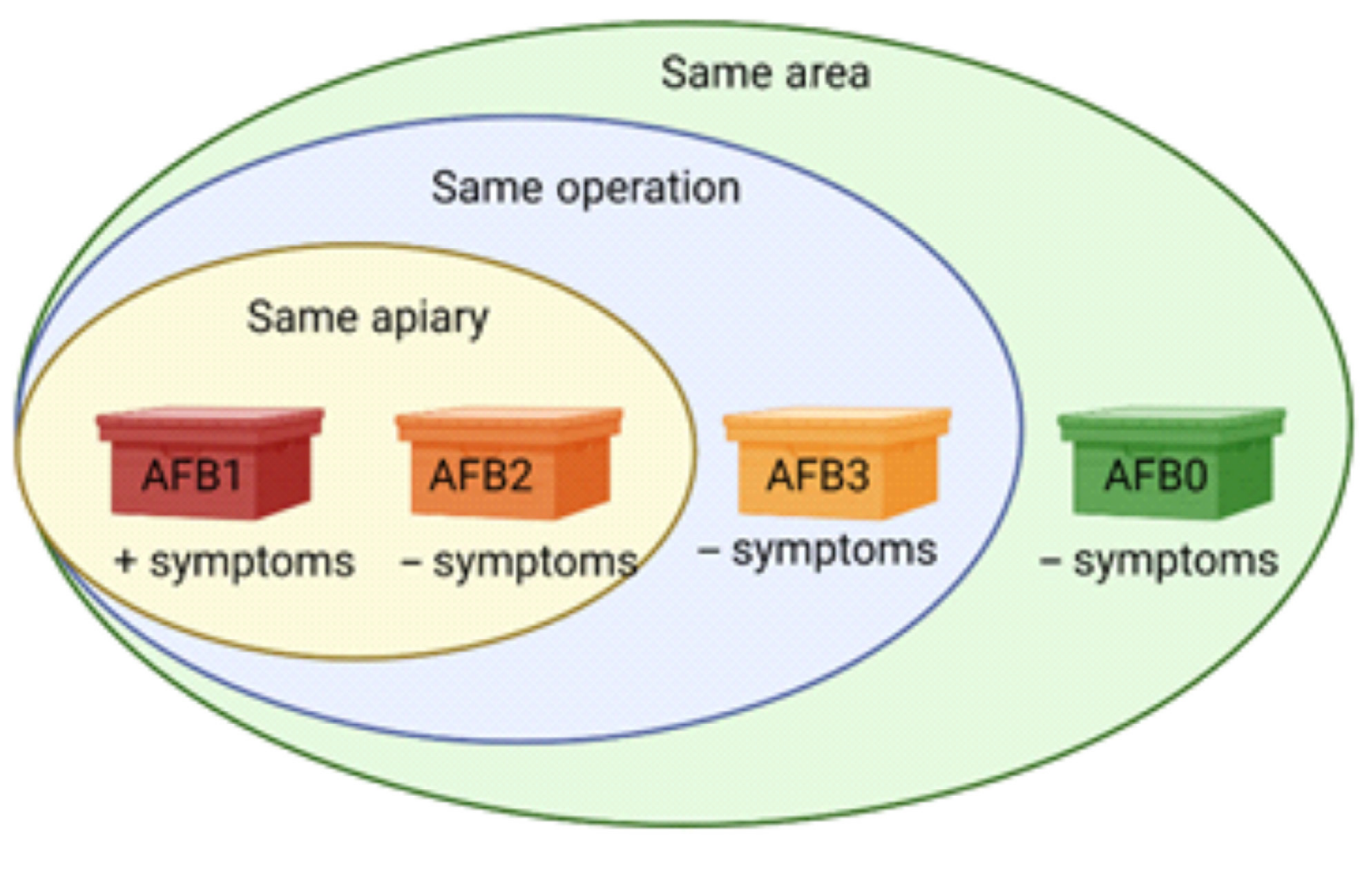
Schematic view over the sample origin.

The samples originated from three geographically separate outbreaks of AFB in Sweden during the summer of 2018. Bee inspectors collected approximately 300 adult honey bees per colony from apiaries in the outbreak areas. The samples were sent to the Swedish National Reference Laboratory for Bee Health and analyzed for *P. larvae* using standard cultivation methods according to Swedish legislation (Forsgren & Laugen, 2014; Nordström & Fries, 1995) (Table 1). Five bees from each 41 samples collected were pooled and used for analyses. The samples were classified according to four categories: honey bee colonies with disease (AFB1, *N* = 3 [one from each separate disease outbreak]), disease‐free colonies from the same apiaries as AFB1 (AFB2, *N* = 9 [3 from each separate disease outbreak]), disease‐free apiaries belonging to beekeeping operations with AFB (AFB3, *N* = 16 [4–6 per outbreak]), and disease‐free beekeeping operations in the AFB‐outbreak areas (AFB0, *N* = 13 [3–5 per outbreak]) (Figure 1, Table 1). Sample information is summarized in Table 1. All honey bee samples were collected during the same period (early May until early June 2018).

**TABLE 1:** Honeybee samples used in this study and information on American foulbrood status for the honey bee colonies the samples were collected from.

Outbreak	Sample	Beekeeper	*Paenibacillus larvae* culture	Visual AFB disease			AFB status category
				Beekeeping operation	Apiary	Colony	
1	1	A	Pos	Yes	Yes	Yes	AFB1
	2	B	Pos	No	No	No	AFB0
	3	B	Neg	No	No	No	AFB0
	4	A	Pos	Yes	No	No	AFB3
	5	A	Neg	Yes	No	No	AFB3
	6	A	Neg	Yes	No	No	AFB3
	7	A	Neg	Yes	No	No	AFB3
	8	A	Pos	Yes	No	No	AFB3
	9	A	Pos	Yes	No	No	AFB3
	10	A	Pos	Yes	Yes	No	AFB2
	11	A	Pos	Yes	Yes	No	AFB2
	12	A	Pos	Yes	Yes	No	AFB2
	13	C	Neg	No	No	No	AFB0
	14	C	Neg	No	No	No	AFB0
	15	C	Neg	No	No	No	AFB0
2	16	D	Pos	Yes	Yes	Yes	AFB1
	17	D	Pos	Yes	Yes	No	AFB2
	18	D	Pos	Yes	Yes	No	AFB2
	19	D	Pos	Yes	Yes	No	AFB2
	20	D	Pos	Yes	No	No	AFB3
	21	D	Neg	Yes	No	No	AFB3
	22	D	Pos	Yes	No	No	AFB3
	23	D	Neg	Yes	No	No	AFB3
	24	D	Pos	Yes	No	No	AFB3
	25	D	Neg	Yes	No	No	AFB3
	26	E	Pos	No	No	No	AFB0
	27	E	Neg	No	No	No	AFB0
	28	E	Pos	No	No	No	AFB0
	29	E	Neg	No	No	No	AFB0
	30	E	Neg	No	No	No	AFB0
3	31	F	Pos	Yes	Yes	Yes	AFB1
	32	F	Pos	Yes	No	No	AFB3
	33	F	Neg	Yes	No	No	AFB3
	34	F	Neg	Yes	No	No	AFB3
	35	F	Neg	Yes	No	No	AFB3
	36	G	Pos	No	No	No	AFB0
	37	G	Neg	No	No	No	AFB0
	38	G	Neg	No	No	No	AFB0
	39	F	Neg	Yes	Yes	No	AFB2
	40	F	Neg	Yes	Yes	No	AFB2
	41	F	Neg	Yes	Yes	No	AFB2

### 2.2 DNA extraction from bee samples

DNA was extracted in duplicates as described by Engel et al. (2013), with minor modifications, using the DNeasy Blood and Tissue‐kit (Qiagen, Hilden, Germany). The bees were washed in a 1% aqueous solution of Klorin Original (Colgate‐Palmolive AB, Stockholm, Sweden) for 2–3 min before being rinsed three times in sterile water. Heads and thoraxes were removed and abdomens were placed in a 2 mL tube (one tube per pooled sample, each pooled sample consisting of five bees) with 5–6 glass beads and 500 μL RLT‐buffer (Qiagen) with beta‐mercaptoethanol (10 μL/mL). The samples were homogenized using Precellys Evolution (Bertin Instruments, Montigny‐le‐Bretonneux, France) with a pre‐set protocol (2 × 5400 rpm for 90 s, 30 s break between runs). The homogenization program was repeated twice and the samples kept on ice between runs. The samples were centrifuged for 2 min at 5000 rpm and 200 μL of the resulting supernatant was mixed with 200 μL 99% ethanol. Two hundred μL of the mixture was used for DNA recovery following the protocol “Purification of Total DNA from Animal Tissues” in the DNeasy Blood and Tissue‐kit (Qiagen, starting at step 4 in DNeasy Blood & Tissue Handbook 07/2006). The DNA was eluted in 60 μL AE‐buffer and the quality and yield of the DNA was estimated using Nanodrop. All DNA samples were diluted to 20 ng/μL and stored at −20°C until analyzed.

### 2.3 DNA extraction from lactic acid bacteria

Ten isolates of hbs‐LAB species (*Lactobacillus apis*, *Apilactobacillus kunkeei*, *Lactobacillus kullabergensis*, *Lactobacillus kimbladii*, *Lactobacillus helsingborgensis*, *Lactobacillus melliventris*, *Apilactobacillus apinorum*, *Bombilactobacillus mellis*, *Bifidobacteria asteroides*, and *Bifidobacteria coryneforme*) (Lamei et al., 2020) were cultured in Man, Rogosa and Sharpe broth (Oxoid, Basingstoke, UK) supplemented with 2% fructose (Merck, Darmstadt, Germany) and 0.1% l‐cysteine (Sigma‐Aldrich, St. Louis, Missouri, USA) (sMRS‐broth) as described by Lamei et al. (2017). Approximately, 10^9^ bacteria of each isolate were harvested and used for DNA extraction. The bacteria were lysed in lysis buffer (20 mM Tris, 2 mM EDTA, 1.2% Triton x‐100) on a shaker at 37°C for 18 h. DNA was purified from the lysate using a QIAcube extraction robot (Qiagen) and the Qiagen DNA extraction protocol “Purification of bacterial or yeast DNA with enzymatic lysis V1,” eluting in 60 μL AE‐buffer (Qiagen). The quality and concentration of the DNA was estimated using Nanodrop.

### 2.4 Assay design

Primers for five *Lactobacilli*, two *Apilactobacilli*, one *Bombilactobacilli*, and two *Bifidobacterium* were designed to fit sequences available through the National Center for Biotechnology Information (NCBI) (Table 2). The primers were designed using the Primer‐BLAST function on NCBI and CLC Main Workbench 8.1 (Qiagen Bioinformatics). The primers were evaluated using qPCR and DNA from reference isolates using SsoFast EvaGreen Supermix (Bio‐Rad) according to the manufacturer's recommendation with an annealing temperature of 60°C followed by a melt curve analysis (hot start 2 min 98°C; 35 cycles 5 s 98°C, 10 s 60°C; melting curve 65–95°C 0.5°C/5 s). To verify the specificity of the primers, the assays were tested with conventional PCR using HotStarTaq Plus DNA Polymerase (Qiagen) according to manufacturer's recommendations. The PCR products were visualized by gel electrophoresis and Sanger‐sequenced (Macrogen Europe, Amsterdam, the Netherlands) to confirm their identity.

**TABLE 2:** Primers used in this study.

Target	Sequence 5′–3′	Reference
*Apilactobacillus apinorum*	CGTGCTGCGAAGGGAATTCCAATTATCAATC	This study
	CAGGGGTTCTCTTTACGGTACCCTTTAGG	
*Lactobacillus apis*	AGGCTGGTTTCACGGTTTACCTGG	This study
	TGTTGGTGTCAACCTGAGAGGCA	
*Lactobacillus helsingborgensis*	CAGATAATGCATTTGTGCGCAGCCTG	This study
	AAAGCTACCGGGACTGTCAGCTACT	
*Lactobacillus kimbladii*	TGGTAAAAGTACAAGCCCACGCC	This study
	TGGAGTCAGTCAGTTCACGAGACAAG	
*Lactobacillus kullabergensis*	GGGCTCTGGTAAAAGTTCAGGCACATG	This study
	GATCTGCCAAGGCTTCTCTAGCCAAA	
*Apilactobacillus kunkeei*	CGATGGTGCCCACATTCAAACAC	This study
	GGAGATGCAGGCAATTGAGCCTTC	
*Bombilactobacillus mellis*	CCACGGTGAAAGGCGACGAAGATATTATG	This study
	TACACCCATTGTTGCGCGTTTAGTTTGAG	
*Lactobacillus melliventris*	TTGGCGGTGTCGGTTTAGGTAAAACA	This study
	GACAACCTTGGCATTAGGCCGTTC	
*Bifidobacterium asteroides*	GAATCCTACCTGTCCTATGCCCTGTC	This study
	TACATGGCGTAGATGACCCTGCG	
*Bifidobacterium coryneforme*	GGATCCGGCGATATTCGAAACAACG	This study
	TCTCCTCATTGGGACGCAGGTC	

### 2.5 qPCR analyses

The qPCR analyses were performed using a Biomark HD system (Fluidigm) following the manufacturer's protocol for gene expression analysis and as described by D'Alvise et al. (2019). To evaluate the quantitative performance of the assays, the assays were tested on FlexSix GE integrated fluidic circuits (IFC) or on 96 × 96 IFCs for gene expression (Fluidigm, San Francisco, CA, USA) using 10‐dilution series of extracted hbs‐LAB DNA as template. Eight to twelve replicates of the assays were matched with a six‐ to eight‐step dilution series of each hbs‐LAB with known DNA concentration to determine detection limit, linear dynamic detection range, variation at detection limits, and PCR efficiency (all above 95%). All assays had a PCR efficiency above 95% and were able to detect as few as 40–850 bacteria per reaction. The final qPCRs were performed on 96 × 96 IFCs for gene expression (Fluidigm) using the manufacturer's standard qPCR protocol for fast PCR followed by a melt curve analysis (thermal mix: 40 min 70°C, 30 s 60°C; hot start: 60 s 95°C; 30 cycles 5 s 96°C, 20 s 60°C; melting curve: 60–95°C 1°C/s). Each duplicate of the extracted DNA was analyzed in triplicates and repeated twice. The qPCR data was checked using the automated quality control in the software Fluidigm Real‐Time PCR analysis and further checked and revised manually. Only Cq‐values derived from reactions that showed a specific melting temperature peak of the product were used in the analysis.

### 2.6 Data analysis and statistics

Logarithmic linear regression was used for the conversion of Cq‐values to number of bacteria per reaction and converted to bacteria per honey bee by back‐calculation of dilutions. Means of the bacterial concentrations per honey bee was determined in duplicate, and was logarithmically (log_10_) transformed for use in the statistical analysis. The diversity of hbs‐LAB species was estimated for each individual sample using the calculated mean bacterial numbers using Shannon's diversity index.

The statistical software SPSS statistics (IBM SPSS Statistics for Windows, Version 26.0) was used for data analysis and significance testing between groups. Normality and homogeneity of variances of the dataset was checked using Shapiro–Wilk's test for normality and Levene's test for homogeneity before the groups' mean values were compared. Since Shannon's diversity index data met the requirements for normality and homogeneity, an ANOVA was used to compare the means of three groups or more (AFB status and outbreak region) and independent *t*‐tests for comparing two groups (visual AFB symptoms in the beekeeping operation, in the apiary, or in the colony, as well as *P. larvae* presence or absence).

The abundance of the respective hbs‐LAB was not normally distributed between individuals. A Kruskal–Wallis with Dunn's post hoc test was therefore used to compare the means of three groups or more (AFB status and outbreak region) and Mann–Whitney *U* test for comparison between two groups (visual AFB symptoms in beekeeping operation, apiary and colony, and *P. larvae* presence or absence).

The program R‐4.2.2 (Innocent and Trusting; R Core Team, 2022) was used for determining community composition differences between AFB groups. A Bray‐Curtis matrix was created on normalized species counts using the vegan package.

## 3 RESULTS

### 3.1 Distribution of honey bee‐specific lactic acid bacteria by outbreak

One honey bee‐specific lactic acid bacterial species (hbs‐LAB), *A. apinorum*, was not detected in any of the samples and was therefore excluded from the statistical analyses (Table 3, Appendix 1). All other hbs‐LAB (*L. apis*, *A. kunkeei*, *L. kullabergensis*, *L. kimbladii*, *L. helsingborgensis*, *L. melliventris*, *B. mellis*, *B. asteroides*, and *B. coryneforme*) were found in all three outbreaks and in all four AFB groups (Table 3, Appendices 1 and 2). Four were detected in all samples: *L. apis*, *L. kullabergensis*, *L. melliventris*, and *B. mellis*, (Table 3, Appendices 1 and 2). Four additional bacterial species were found in a majority of the samples: *L. helsingborgensis*, *L. kimbladii*, *B. asteroides*, and *B. coryneforme* (97.6%, 85.4%, 95.1%, and 68.3% respectively; Table 3, Appendix 1). There were some differences in distribution between outbreaks; *L. helsingborgensis* and *B. asteroides* were found in all samples (AFB0–AFB3) from outbreaks 1 and 2, but for outbreak 3 one AFB0 sample was negative for *L. helsingborgensis*, and one AFB 1 sample and one AFB 2 sample were negative for *B. asteroids* (Table 3, Appendix 1). Conversely, *L. kimbladii* was found in all samples from outbreak 3 but was not present in one sample from outbreak 1 and 5 samples from outbreak 2. *B. coryneforme* was found in all but one sample from outbreak 1 (93.3%) compared to in eight (53.3%) and six (54.5%) from outbreaks 2 and 3, respectively (Table 3, Appendix 1). *A. kunkeei* was detected in 12 (29.3%) of the overall samples and only in one sample from outbreak 3 (40% of outbreak 1 samples and 33.3% of outbreak 2 samples) (Table 3, Appendix 1).

**TABLE 3:** The distribution of honeybee‐specific lactic acid bacteria in 41 honeybee samples.

Hbs‐LAB	Outbreak			AFB status				Total
	1	2	3	AFB1	AFB2	AFB3	AFB0	
	*n* = 15	*n* = 15	*n* = 11	*n* = 3	*n* = 9	*n* = 16	*n* = 13	*n* = 41
	(%)	(%)	(%)	(%)	(%)	(%)	(%)	(%)
*Lactobacillus apis*	15	15	11	3	9	16	13	41
	(100)	(100)	(100)	(100)	(100)	(100)	(100)	(100)
*Apilactobacillus kunkeei*	6	5	1	1	5	3	3	12
	(40)	(33.3)	(9.1)	(33.3)	(55.6)	(18.8)	(23.1)	(29.3)
*Lactobacillus kullabergensis*	15	15	11	3	9	16	13	41
	(100)	(100)	(100)	(100)	(100)	(100)	(100)	(100)
*Lactobacillus kimbladii*	14	10	11	2	7	14	12	35
	(93.3)	(66.7)	(100)	(66.7)	(77.8)	(87.5)	(92.3)	(85.4)
*Lactobacillus helsingborgensis*	15	15	10	3	9	16	12	40
	(100)	(100)	(90.9)	(100)	(100)	(100)	(92.3)	(97.6)
*Lactobacillus melliventris*	15	15	11	3	9	16	13	41
	(100)	(100)	(100)	(100)	(100)	(100)	(100)	(100)
*Bombilactobacillus mellis*	15	15	11	3	9	16	13	41
	(100)	(100)	(100)	(100)	(100)	(100)	(100)	(100)
*Apilactobacillus apinorum*	0	0	0	0	0	0	0	0
	(0)	(0)	(0)	(0)	(0)	(0)	(0)	(0)
*Bifidobacteria asteroides*	15	15	9	2	8	16	13	39
	(100)	(100)	(81.8)	(66.7)	(88.9)	(100)	(100)	(95.1)
*Bifidobacteria coryneforme*	14	8	6	2	5	11	10	28
	(93.3)	(53.3)	(54.5)	(66.7)	(55.6)	(68.8)	(76.9)	(68.3)

*Note*: The table shows the distribution per outbreak and according to AFB status.

### 3.2 Abundance of hbs‐LABs

**FIGURE 2:**
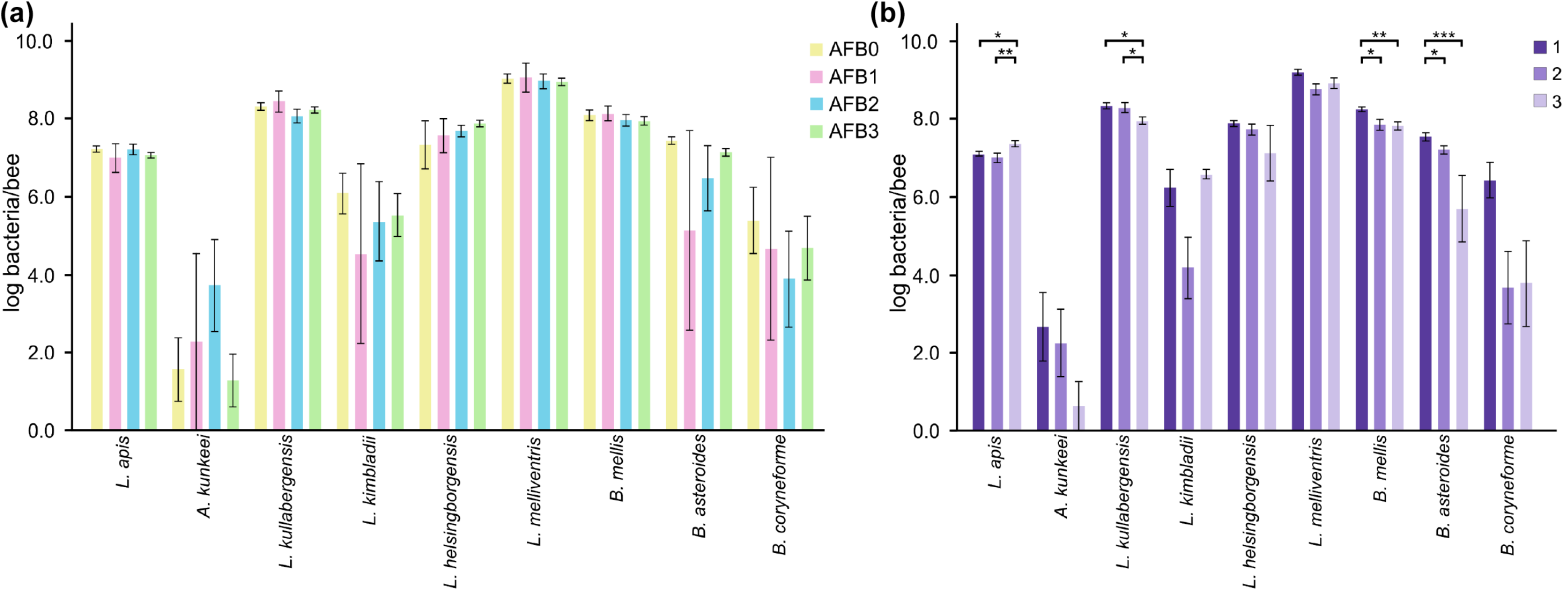
Abundance of bacteria found in honeybees collected during outbreaks of AFB in Sweden 2018. The graph shows the mean of the log10 bacteria/bee concentration and standard error of mean for each group regarding AFB status (a) and outbreak (b). *Apilactobacillus apinorum* is not included in the figure as all samples were negative. Significant differences are indicated. *p < .05; **p < .01; ***p < .001.

The abundance of hbs‐LABs was compared between different AFB status groups as well as different outbreaks. While no significant differences were found between the different AFB status groups (Figure 2a), a Kruskal‐Wallis comparison of samples from the three outbreaks revealed differences in four bacterial species: *L. apis* (*p* = .019), *L. kullabergensis* (*p* = .030), *B. mellis* (*p* = .008), and *B. asteroides* (*p* < .001). The post hoc analysis revealed that samples from outbreak 1 had higher abundance of *B. mellis* and *B. asteroides* than samples from outbreak 2 (*p* = .015 and .049 respectively) (Figure 2b) and a higher abundance of *L. kullabergensis*, *B. mellis*, and *B. asteroides* (*p* = .013, *p* = .004 and *p* < .001, respectively) than samples from outbreak 3 (Figure 2b). The abundance of *L. kullabergensis* was significantly higher in honey bees from outbreak 2 than in 3 (*p* = .030, respectively) (Figure 2b). Samples from outbreak 3 had a higher abundance of *L. apis* than both outbreak 1 and 2 (*p* = .032 and *p* = .007, respectively) (Figure 2b). Additionally, a significantly higher abundance of *B. asteroides* was found in samples from beekeeping operations with colonies without visible AFB symptoms (*n* = 13, *p* = .041, data not shown).

Overall, the community composition of the different bacterial communities was different between AFB groups. The group with visible AFB symptoms (AFB1) was moderately similar to the other three groups (Bray–Curtis dissimilarity index 0.52–0.70, Table 4), while the other three groups (AFB0, AFB2, and AFB3) were not very similar to each other (Bray Curtis dissimilarity index 0.1–0.27, Table 4).

**TABLE 4:** Bray–Curtis dissimilarity index for the AFB groups.

Category	AFB1	AFB0	AFB3
AFB0	0.6406250	–	0.1025641
AFB3	0.6973684	0.1025641	–
AFB2	0.5257732	0.1731844	0.2709360

*Note*: Blue are similar and red are not similar.

### 3.3 Diversity of hbs‐LAB

**FIGURE 3:**
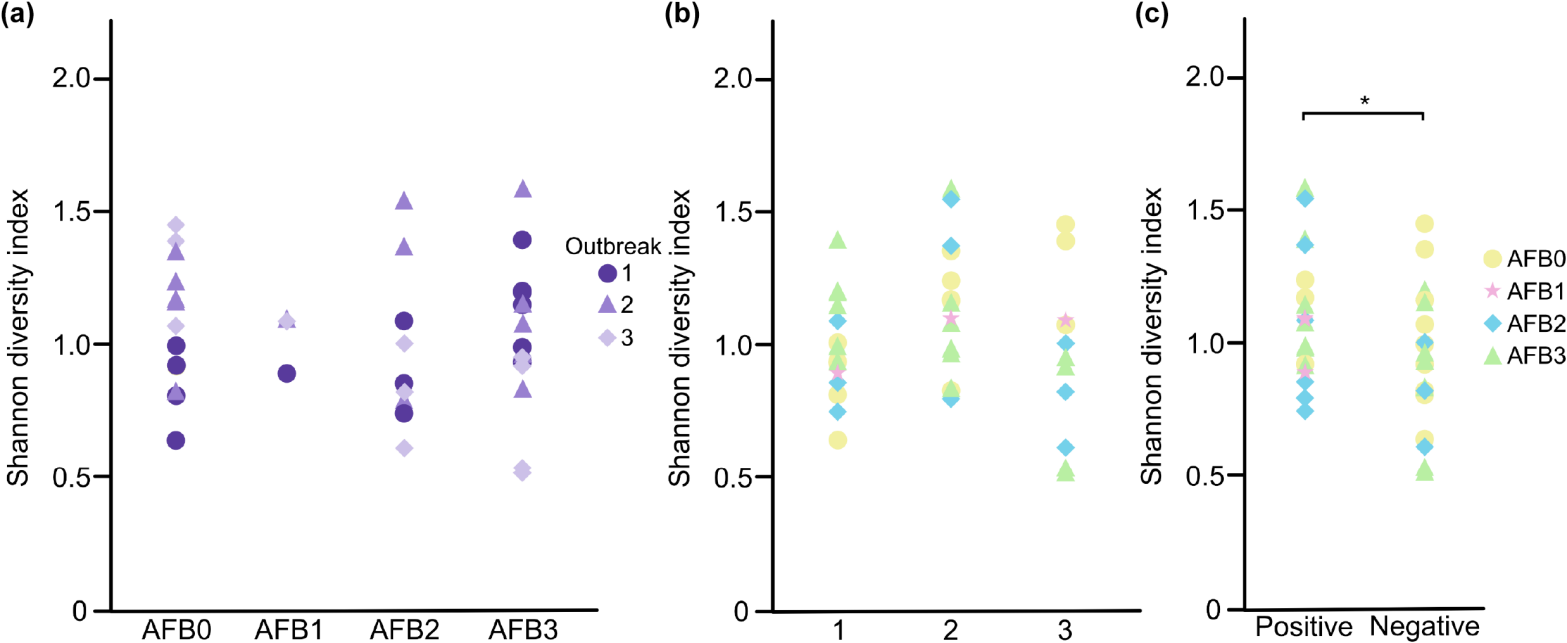
Hbs‐LAB diversity (Shannon diversity index) in samples from colonies regarding AFB status (a), outbreak region (b), and *Paenibacillus larvae* culture result (c). Significant differences are indicated. *p < .05.

A comparison of the diversity between samples from *P. larvae* culture‐positive (*n* = 20, Tables 1 and 5) and culture‐negative (*n* = 21, Tables 1 and 5) colonies revealed that the mean Shannon's diversity index for the hbs‐LAB was higher in culture‐positive samples (*p* = .040, Figure 3c). However, no difference in diversity, as measured by Shannon's diversity index, was identified between sample groups when the three outbreaks and the different AFB status were compared (Figure 3a,b). There was almost no difference in community composition between the samples that were *P. larvae* positive compared to those that were not.

**TABLE 5:** Proportions of samples positive for the pathogen *Paenibacillus larvae* by outbreak and AFB status.

Outbreak	AFB1	AFB2	AFB3	AFB0	Total
1	100%	100%	50%	20%	53.3%
	(1/1)	(3/3)	(3/6)	(1/5)	(8/15)
2	100%	100%	50%	40%	60%
	(1/1)	(3/3)	(3/6)	(2/5)	(9/15)
3	100%	0%	25%	33.3%	27.3%
	(1/1)	(0/3)	(1/4)	(1/3)	(3/11)
Total	100%	66.7%	70%	30.8%	48.8%
	(3/3)	(6/9)	(7/10)	(4/13)	(20/41)

## 4 DISCUSSION

Forty‐one samples collected from three AFB outbreaks in Sweden (2018) were assayed for ten hbs‐LAB species. Our results show that four of the assayed hbs‐LAB species were present in all samples while one of the hbs‐LAB, *A. apinorum*, was not detected in any of the samples. Changes in the microbiome have been reported in association with pathogen pressure, not only with *P. larvae* (Erban, Ledvinka, Kamler, Nesvorna, et al., 2017; Lamei et al., 2020; Ye et al., 2021), but also in association with Varroa infestations, Vairimorpha (previously Nosema) infection, neogregarine infections, and the bacteria *Melissococcus plutonius* that causes European foulbrood (Erban, Ledvinka, Kamler, Hortova, et al., 2017; Hubert et al., 2017; Ptaszyńska et al., 2021). This suggests that even though adult honey bees are not directly affected by the diseases caused by the pathogens, the hbs‐LAB may be a factor or play a role in the course of honey bee brood diseases. This may happen by alteration of the hygienic behavior of the adult honey bees or by altering the bacterial microbiome of honey bee brood through the feeding of the larvae. Although we did not find any overall differences in hbs‐LAB abundance or diversity in honey bees from symptomatic or asymptomatic colonies, we did find a higher diversity of hbs‐LAB in individuals from bee colonies with *P. larvae*. This is similar to the results in Lamei et al. (2020) where the hbs‐LAB diversity was higher in bee samples from colonies with an AFB history compared to colonies without any history of AFB. In addition to differences in diversity, we found differences in the abundance of the individual hbs‐LABs between outbreaks. This is contrary to studies on the microbiota of humans and mice where a lower diversity was found in individuals infected with bacterial enteropathogens compared to uninfected individuals (Kampmann et al., 2016; Stecher et al., 2010). However, as our background knowledge of the colonies, apiaries, and beekeeping operations in our study is limited, we cannot conclude that AFB or the presence of *P. larvae* exclusively causes these differences. Several other factors such as age and quality of equipment, microclimate, foraging resources, or pathogen pressure may have an effect on the abundance and the prevalence of each individual hbs‐LAB. The samples included in this study were collected from three AFB outbreaks in different geographic locations; hence, both microclimate and foraging resources might have had an impact on the composition of the hbs‐LAB species in this study. *A. kunkeei* has been suggested to be the most common and abundant species in the hbs‐LAB microbiota (Vásquez et al., 2009, 2012). However, in this study, *L. melliventris* was the most abundant hbs‐LAB in all but one sample where the most abundant and equally numerous bacteria were *L. melliventris* and *L. kullabergensis* (Appendix 2). Studies where *A. kunkeei* has been found to be the most abundant hbs‐LAB are, however, based on analyses of DNA extracted from cultured bacteria. This means that the estimations may be biased due to variation in growth rate and ability to grow in culture media. This is supported by other studies where they have used culture‐independent methods and found little or no *A. kunkeei* in honey bees (Engel et al., 2012; Martinson et al., 2011; Moran et al., 2012; Sabree et al., 2012) and by Lamei et al. (2017), who compared growth rates of hbs‐LABs and showed that *A. kunkeei* and *A. apinorum* had higher growth rates in artificial media compared to other hbs‐LAB. This is a plausible explanation for the high abundance of *A. kunkeei* reported from culture‐based studies. In this study, where DNA was extracted directly from the bee guts, *A. kunkeei* was prevalent in 29.3% of all the samples and only in 1 of 11 samples (9%) from one of the outbreaks (outbreak 3). This is in line with similar studies where they have shown that the relative abundance of *A. kunkeei* in the hbs‐LAB is affected by seasonal changes and food resources (Olofsson & Vásquez, 2008; Seeburger et al., 2020). The abundance of *A. kunkeei* may also vary depending on the diet of the honey bees. In one study where honey bees were fed a diet mimicking honeydew, a decrease in *A. kunkeei* was seen when compared to a control group fed a diet consisting of glucose, sucrose, and fructose (Seeburger et al., 2020). Furthermore, an increased abundance of *A. kunkeei* has been observed in bees collecting nectar from linden flowers (Olofsson & Vásquez, 2008). The proportion of *A. kunkeei* in the honey bee microbiome has been shown to be significantly higher in colonies with EFB than in healthy colonies (Erban, Ledvinka, Kamler, Hortova, et al., 2017). Interestingly, a lower pathogen load and pathogen activity has been shown in honey bees fed pollen patties supplemented with *Lactobacillus rhamnosus*, *Lactiplantibacillus plantarum*, and *A. kunkeei* compared to untreated bees and bees fed pollen patties without supplement of bacteria during an AFB outbreak (Daisley et al., 2020). However, other studies show that positive effects from lactobacilli supplements on individual bees not automatically translates to colony‐level effects, and it may be critical how the lactobacilli are administrated (Daisley et al., 2020, 2023; Lamei et al., 2020; Stephan et al., 2019). Further studies where bees are monitored over a longer period and data on variations in foraging sources, climate, etc. are needed to better understand how environmental factors may affect the composition of hbs‐LAB and how this affects honey bee health and their susceptibility to infections.

A negative correlation between *P. larvae* and the hbs‐LABs *B. asteroides* and *B. mellis* in honey bees have previously been reported (Erban, Ledvinka, Kamler, Nesvorna, et al., 2017), and we observed a higher abundance of *B. asteroides* in the honey bees from beekeeping operations free from colonies with visual AFB signs. The production of prostaglandins in honey bees have been shown to be induced by *B. asteroides* (Kešnerová et al., 2017) and to be involved in fluid secretion, reproduction, and activation of the immune system in insects (Stanley & Kim, 2011). Though the function of the *B. asteroides* induced prostaglandins in honey bees have not been fully studied (Kešnerová et al., 2017), they may play a role in honey bees protection against infections by activating the immune system and may explain the higher abundance of *B. asteroides* in beekeeping operations without AFB.

In conclusion, we found no differences in the abundance or diversity of the hbs‐LAB composition between colonies with or without AFB. However, we observed a higher diversity of hbs‐LAB in honey bees from colonies where *P. larvae* had been detected and saw differences in the abundance of the individual hbs‐LAB in honey bees from different AFB outbreaks. Longitudinal studies, in which the health status of the bees, their microbiota, and climate and foraging resources data are monitored over longer times, are needed for a better understanding of what affects honey bee health and susceptibility to diseases.

## AUTHOR CONTRIBUTIONS

**Anna Nilsson:** Conceptualization (equal); data curation (lead); formal analysis (lead); methodology (equal); visualization (lead); writing – original draft (lead); writing – review and editing (equal). **Paul D'Alvise:** Conceptualization (equal); formal analysis (supporting); methodology (equal); writing – review and editing (equal). **Meghan O. Milbrath:** Formal analysis (supporting); visualization (supporting); writing – review and editing (equal). **Eva Forsgren:** Conceptualization (equal); methodology (equal); resources (lead); writing – review and editing (equal).

## CONFLICT OF INTEREST STATEMENT

We declare that there are no conflicts of interest regarding the article.

## Figures and Tables

**Figure:**
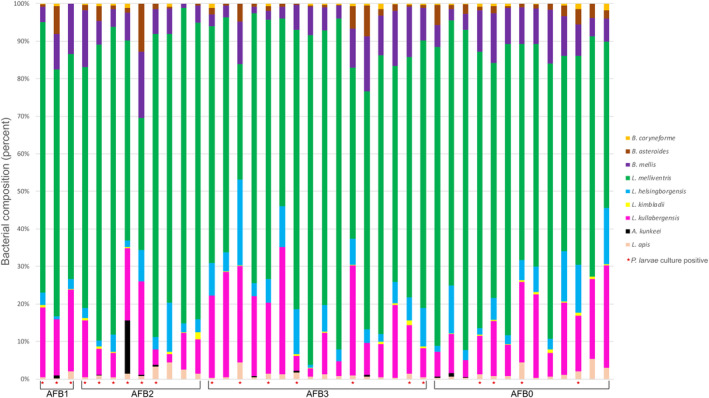
Hbs‐LAB abundance for the individual samples. All samples were negative for *Apilactobacillus apinorum* and the results for *A. apinorum* are not included in the figure.

## Data Availability

All qPCR results (Cq‐value table) have been submitted to the Swedish National Data Service and will be published upon acceptance of the manuscript. The data can be accessed by the accession https://doi.org/10.5878/kbt4‐b102.

## APPENDIX 1

qPCR result for hbs‐LAB shown as positive (blue) or negative (red) for each individual honeybee sample.

**Table:**

Outbreak	Sample	Hbs‐LAB qPCR									
		*Lactobacillus apis*	*Apilactobacillus kunkeei*	*Lactobacillus kullabergensis*	*Lactobacillus kimbladii*	*Lactobacillus helsingborgensis*	*Lactobacillus melliventris*	*Apilactobacillus apinorum*	*Bombilactobacillus mellis*	*Bifidobacteria asteroides*	*Bifidobacteria coryneforme*
1	1	+	−	+	+	+	+	−	+	+	+
	2	+	−	+	+	+	+	−	+	+	+
	3	+	−	+	+	+	+	−	+	+	−
	4	+	−	+	+	+	+	−	+	+	+
	5	+	+	+	+	+	+	−	+	+	+
	6	+	−	+	+	+	+	−	+	+	+
	7	+	−	+	+	+	+	−	+	+	+
	8	+	−	+	+	+	+	−	+	+	+
	9	+	−	+	+	+	+	−	+	+	+
	10	+	−	+	+	+	+	−	+	+	+
	11	+	+	+	+	+	+	−	+	+	+
	12	+	+	+	+	+	+	−	+	+	+
	13	+	+	+	+	+	+	−	+	+	+
	14	+	+	+	+	+	+	−	+	+	+
	15	+	+	+	−	+	+	−	+	+	+
2	16	+	+	+	−	+	+	−	+	+	+
	17	+	+	+	+	+	+	−	+	+	+
	18	+	+	+	−	+	+	−	+	+	−
	19	+	+	+	−	+	+	−	+	+	−
	20	+	−	+	−	+	+	−	+	+	+
	21	+	−	+	+	+	+	−	+	+	−
	22	+	−	+	+	+	+	−	+	+	−
	23	+	+	+	+	+	+	−	+	+	−
	24	+	−	+	+	+	+	−	+	+	+
	25	+	−	+	−	+	+	−	+	+	−
	26	+	−	+	+	+	+	−	+	+	+
	27	+	−	+	+	+	+	−	+	+	+
	28	+	−	+	+	+	+	−	+	+	−
	29	+	−	+	+	+	+	−	+	+	+
	30	+	−	+	+	+	+	−	+	+	+
3	31	+	−	+	+	+	+	−	+	−	−
	32	+	+	+	+	+	+	−	+	+	−
	33	+	−	+	+	+	+	−	+	+	+
	34	+	−	+	+	+	+	−	+	+	+
	35	+	−	+	+	+	+	−	+	+	+
	36	+	−	+	+	+	+	−	+	+	+
	37	+	−	+	+	−	+	−	+	+	−
	38	+	−	+	+	+	+	−	+	+	+
	39	+	−	+	+	+	+	−	+	+	+
	40	+	−	+	+	+	+	−	+	−	−
	41	+	−	+	+	+	+	−	+	+	−
Total pos		41	12	41	35	40	41	0	41	39	28
% Pos		100.0	29.3	100.0	85.4	97.6	100.0	0.0	100.0	95.1	68.3

## References

[ece310964-bib-0001] Aizen, M. A. , & Harder, L. D. (2009). The global stock of domesticated honey bees is growing slower than agricultural demand for pollination. Current Biology, 19, 915–918. 10.1016/j.cub.2009.03.071 19427214

[ece310964-bib-0002] Corby‐Harris, V. , Maes, P. , & Anderson, K. E. (2014). The bacterial communities associated with honey bee (*Apis mellifera*) foragers. PLoS One, 9, e95056. 10.1371/journal.pone.0095056 24740297 PMC3989306

[ece310964-bib-0003] Cox‐Foster, D. L. , Conlan, S. , Holmes, E. C. , Palacios, G. , Evans, J. D. , Moran, N. A. , Quan, P.‐L. , Briese, T. , Hornig, M. , Geiser, D. M. , Martinson, V. , vanEngelsdorp, D. , Kalkstein, A. L. , Drysdale, A. , Hui, J. , Zhai, J. , Cui, L. , Hutchison, S. K. , Simons, J. F. , … Lipkin, W. I. (2007). A metagenomic survey of microbes in honey bee colony collapse disorder. Science, 318, 283–287. 10.1126/science.1146498 17823314

[ece310964-bib-0004] Crailsheim, K. , & Riessberger‐Gallé, U. (2001). Honey bee age‐dependent resistance against American foulbrood. Apidologie, 32, 13–103.

[ece310964-bib-0005] Daisley, B. A. , Pitek, A. P. , Chmiel, J. A. , Al, K. F. , Chernyshova, A. M. , Faragalla, K. M. , Burton, J. P. , Thompson, G. J. , & Reid, G. (2020). Novel probiotic approach to counter *Paenibacillus larvae* infection in honey bees. The ISME Journal, 14, 476–491. 10.1038/s41396-019-0541-6 31664160 PMC6976702

[ece310964-bib-0006] Daisley, B. A. , Pitek, A. P. , Mallory, E. , Chernyshova, A. M. , Allen‐Vercoe, E. , Reid, G. , & Thompson, G. J. (2023). Disentangling the microbial ecological factors impacting honey bee susceptibility to *Paenibacillus larvae* infection. Trends in Microbiology, 31, 521–534. 10.1016/j.tim.2022.11.012 36526535

[ece310964-bib-0007] D'Alvise, P. , Seeburger, V. , Gihring, K. , Kieboom, M. , & Hasselmann, M. (2019). Seasonal dynamics and co‐occurrence patterns of honey bee pathogens revealed by high‐throughput RT‐qPCR analysis. Ecology and Evolution, 9, 10241–10252. 10.1002/ece3.5544 31624548 PMC6787843

[ece310964-bib-0008] Engel, P. , James, R. R. , Koga, R. , Kwong, W. K. , McFrederick, Q. S. , & Moran, N. A. (2013). Standard methods for research on *Apis mellifera* gut symbionts. Journal of Apicultural Research, 52, 1–24. 10.3896/IBRA.1.52.4.07

[ece310964-bib-0009] Engel, P. , Kwong, W. K. , McFrederick, Q. , Anderson, K. E. , Barribeau, S. M. , Chandler, J. A. , Cornman, R. S. , Dainat, J. , de Miranda, J. R. , Doublet, V. , Emery, O. , Evans, J. D. , Farinelli, L. , Flenniken, M. L. , Granberg, F. , Grasis, J. A. , Gauthier, L. , Hayer, J. , Koch, H. , … Dainat, B. (2016). The bee microbiome: Impact on bee health and model for evolution and ecology of host‐microbe interactions. MBio, 7(2), e02164‐15. 10.1128/mBio.02164-15 27118586 PMC4850275

[ece310964-bib-0010] Engel, P. , Martinson, V. G. , & Moran, N. A. (2012). Functional diversity within the simple gut microbiota of the honey bee. Proceedings of the National Academy of Sciences of the United States of America, 109, 11002–11007. 10.1073/pnas.1202970109 22711827 PMC3390884

[ece310964-bib-0011] Erban, T. , Ledvinka, O. , Kamler, M. , Hortova, B. , Nesvorna, M. , Tyl, J. , Titera, D. , Markovic, M. , & Hubert, J. (2017). Bacterial community associated with worker honeybees (*Apis mellifera*) affected by European foulbrood. PeerJ, 5, e3816. 10.7717/peerj.3816 28966892 PMC5619233

[ece310964-bib-0012] Erban, T. , Ledvinka, O. , Kamler, M. , Nesvorna, M. , Hortova, B. , Tyl, J. , Titera, D. , Markovic, M. , & Hubert, J. (2017). Honeybee (*Apis mellifera*)‐associated bacterial community affected by American foulbrood: Detection of *Paenibacillus larvae* via microbiome analysis. Scientific Reports, 7, 5084. 10.1038/s41598-017-05076-8 28698604 PMC5506040

[ece310964-bib-0013] Evans, J. D. , & Lopez, D. L. (2004). Bacterial probiotics induce an immune response in the honey bee (Hymenoptera: Apidae). Journal of Economic Entomology, 97, 752–756. 10.1603/0022-0493(2004)097[0752:bpiair]2.0.co;2 15279248

[ece310964-bib-0014] Forsgren, E. , & Laugen, A. T. (2014). Prognostic value of using bee and hive debris samples for the detection of American foulbrood disease in honey bee colonies. Apidologie, 45, 10–20. 10.1007/s13592-013-0225-6

[ece310964-bib-0015] Forsgren, E. , Olofsson, T. C. , Vásquez, A. , & Fries, I. (2010). Novel lactic acid bacteria inhibiting *Paenibacillus larvae* in honey bee larvae. Apidologie, 41, 99–108. 10.1051/apido/2009065

[ece310964-bib-0016] Gallai, N. , Salles, J.‐M. , Settele, J. , & Vaissière, B. E. (2009). Economic valuation of the vulnerability of world agriculture confronted with pollinator decline. Ecological Economics, 68, 810–821. 10.1016/j.ecolecon.2008.06.014

[ece310964-bib-0017] Genersch, E. (2010a). Honey bee pathology: Current threats to honey bees and beekeeping. Applied Microbiology and Biotechnology, 87, 87–97. 10.1007/s00253-010-2573-8 20401479

[ece310964-bib-0018] Genersch, E. (2010b). American foulbrood in honeybees and its causative agent, *Paenibacillus larvae* . Journal of Invertebrate Pathology, 103, S10–S19. 10.1016/j.jip.2009.06.015 19909971

[ece310964-bib-0019] Goulson, D. , Nicholls, E. , Botias, C. , & Rotheray, E. L. (2015). Bee declines driven by combined stress from parasites, pesticides, and lack of flowers. Science, 347, 1255957. 10.1126/science.1255957 25721506

[ece310964-bib-0020] Hubert, J. , Bicianova, M. , Ledvinka, O. , Kamler, M. , Lester, P. J. , Nesvorna, M. , Kopecky, J. , & Erban, T. (2017). Changes in the bacteriome of honey bees associated with the parasite *Varroa destructor*, and pathogens *Nosema* and *Lotmaria passim* . Microbial Ecology, 73, 685–698. 10.1007/s00248-016-0869-7 27730366

[ece310964-bib-0021] Janashia, I. , & Alaux, C. (2016). Specific immune stimulation by endogenous bacteria in honey bees (Hymenoptera: Apidae). Journal of Economic Entomology, 109, 1474–1477. 10.1093/jee/tow065 27063842

[ece310964-bib-0022] Kampmann, C. , Dicksved, J. , Engstrand, L. , & Rautelin, H. (2016). Composition of human faecal microbiota in resistance to campylobacter infection. Clinical Microbiology and Infection, 22, 61.e1–8. 10.1016/j.cmi.2015.09.004 26369602

[ece310964-bib-0023] Kešnerová, L. , Mars, R. A. T. , Ellegaard, K. M. , Troilo, M. , Sauer, U. , & Engel, P. (2017). Disentangling metabolic functions of bacteria in the honey bee gut. PLoS Biology, 15, e2003467. 10.1371/journal.pbio.2003467 29232373 PMC5726620

[ece310964-bib-0024] Khan, K. A. , Al‐Ghamdi, A. A. , Ghramh, H. A. , Ansari, M. J. , Ali, H. , Alamri, S. A. , Al‐ Kahtani, S. N. , Adgaba, N. , Qasim, M. , & Hafeez, M. (2020). Structural diversity and functional variability of gut microbial communities associated with honey bees. Microbial Pathogenesis, 138, 103793. 10.1016/j.micpath.2019.103793 31626917

[ece310964-bib-0025] Killer, J. , Dubná, S. , Sedláček, I. , & Švec, P. (2014). Lactobacillus apis sp. nov., from the stomach of honeybees (*Apis mellifera*), having an in vitro inhibitory effect on the causative agents of American and European foulbrood. International Journal of Systematic and Evolutionary Microbiology, 64, 152–157. 10.1099/ijs.0.053033-0 24096349

[ece310964-bib-0026] Koh, I. , Lonsdorf, E. V. , Williams, N. M. , Brittain, C. , Isaacs, R. , Gibbs, J. , & Ricketts, T. H. (2016). Modeling the status, trends, and impacts of wild bee abundance in the United States. Proceedings of the National Academy of Sciences of the United States of America, 113, 140–145. 10.1073/pnas.1517685113 26699460 PMC4711882

[ece310964-bib-0027] Lamei, S. , Hu, Y. O. O. , Olofsson, T. C. , Andersson, A. F. , Forsgren, E. , & Vásquez, A. (2017). Improvement of identification methods for honeybee specific lactic acid bacteria; future approaches. PLoS One, 12, e0174614. 10.1371/journal.pone.0174614 28346815 PMC5367889

[ece310964-bib-0028] Lamei, S. , Stephan, J. G. , Nilson, B. , Sieuwerts, S. , Riesbeck, K. , de Miranda, J. R. , & Forsgren, E. (2020). Feeding honeybee colonies with honeybee‐specific lactic acid bacteria (Hbs‐LAB) does not affect colony‐level Hbs‐LAB composition or *Paenibacillus larvae* spore levels, although American foulbrood affected colonies harbor a more diverse Hbs‐LAB community. Microbial Ecology, 79, 743–755. 10.1007/s00248-019-01434-3 31506760 PMC7176604

[ece310964-bib-0029] Martinson, V. G. , Danforth, B. N. , Minckley, R. L. , Rueppell, O. , Tingek, S. , & Moran, N. A. (2011). A simple and distinctive microbiota associated with honey bees and bumble bees. Molecular Ecology, 20, 619–628. 10.1111/j.1365-294X.2010.04959.x 21175905

[ece310964-bib-0030] Moran, N. A. , Hansen, A. K. , Powell, J. E. , & Sabree, Z. L. (2012). Distinctive gut microbiota of honey bees assessed using deep sampling from individual worker bees. PLoS One, 7, e36393. 10.1371/journal.pone.0036393 22558460 PMC3338667

[ece310964-bib-0031] Neumann, P. , & Carreck, N. L. (2010). Honey bee colony losses. Journal of Apicultural Research, 49, 1–6. 10.3896/IBRA.1.49.1.01

[ece310964-bib-0032] Nordström, S. , & Fries, I. (1995). A comparison of media and cultural conditions for identification of *Bacillus larvae* in honey. Journal of Apicultural Research, 34, 97–103. 10.1080/00218839.1995.11100894

[ece310964-bib-0033] Olofsson, T. C. , Alsterfjord, M. , Nilson, B. , Butler, È. , & Vásquez, A. (2014). *Lactobacillus apinorum* sp. nov., *Lactobacillus mellifer* sp. nov., *Lactobacillus mellis* sp. nov., *Lactobacillus melliventris* sp. nov., *Lactobacillus kimbladii* sp. nov., *Lactobacillus helsingborgensis* sp. nov. and *Lactobacillus kullabergensis* sp. nov., isolated from the honey stomach of the honeybee *Apis mellifera* . International Journal of Systematic and Evolutionary Microbiology, 64, 3109–3119. 10.1099/ijs.0.059600-0 24944337 PMC4156108

[ece310964-bib-0034] Olofsson, T. C. , Butler, È. , Markowicz, P. , Lindholm, C. , Larsson, L. , & Vásquez, A. (2016). Lactic acid bacterial symbionts in honeybees – An unknown key to honey's antimicrobial and therapeutic activities. International Wound Journal, 13, 668–679. 10.1111/iwj.12345 25195876 PMC7949542

[ece310964-bib-0035] Olofsson, T. C. , & Vásquez, A. (2008). Detection and identification of a novel lactic acid bacterial flora within the honey stomach of the honeybee *Apis mellifera* . Current Microbiology, 57, 356–363. 10.1007/s00284-008-9202-0 18663527

[ece310964-bib-0036] Pachla, A. , Wicha, M. , Ptaszyńska, A. A. , Borsuk, G. , Trokenheim, Ł. Ł. , & Małek, W. (2018). The molecular and phenotypic characterization of fructophilic lactic acid bacteria isolated from the guts of *Apis mellifera* L. derived from a polish apiary. Journal of Applied Genetics, 59, 503–514. 10.1007/s13353-018-0467-0 30269313

[ece310964-bib-0037] Pătruică, S. , & Mot, D. (2012). The effect of using prebiotic and probiotic products on intestinal micro‐flora of the honeybee (*Apis mellifera carpatica*). Bulletin of Entomological Research, 102, 619–623. 10.1017/S0007485312000144 22459313

[ece310964-bib-0038] Ptaszyńska, A. A. , Latoch, P. , Hurd, P. J. , Polaszek, A. , Michalska‐Madej, J. , Grochowalski, Ł. , Strapagiel, D. , Gnat, S. , Załuski, D. , Gancarz, M. , Rusinek, R. , Krutmuang, P. , Martín Hernández, R. , Higes Pascual, M. , & Starosta, A. L. (2021). Amplicon sequencing of variable 16S rRNA from bacteria and ITS2 regions from fungi and plants, reveals honeybee susceptibility to diseases results from their forage availability under anthropogenic landscapes. Pathogens, 10, 381. 10.3390/pathogens10030381 33810160 PMC8004708

[ece310964-bib-0039] R Core Team . (2022). R: A language and environment for statistical computing. R Foundation for Statistical Computing. https://www.R‐project.org/

[ece310964-bib-0040] Raymann, K. , & Moran, N. A. (2018). The role of the gut microbiome in health and disease of adult honey bee workers. Current Opinion in Insect Science, 26, 97–104. 10.1016/j.cois.2018.02.012 29764668 PMC6010230

[ece310964-bib-0041] Sabree, Z. L. , Hansen, A. K. , & Moran, N. A. (2012). Independent studies using deep sequencing resolve the same set of core bacterial species dominating gut communities of honey bees. PLoS One, 7, e41250. 10.1371/journal.pone.0041250 22829932 PMC3400611

[ece310964-bib-0042] Seeburger, V. C. , D'Alvise, P. , Shaaban, B. , Schweikert, K. , Lohaus, G. , Schroeder, A. , & Hasselmann, M. (2020). The trisaccharide melezitose impacts honey bees and their intestinal microbiota. PLoS One, 15, e0230871. 10.1371/journal.pone.0230871 32275718 PMC7147780

[ece310964-bib-0043] Stanley, D. , & Kim, Y. (2011). Prostaglandins and their receptors in insect biology. Frontiers in Endocrinology, 2, 105. 10.3389/fendo.2011.00105 22654840 PMC3356066

[ece310964-bib-0044] Stecher, B. , Chaffron, S. , Käppeli, R. , Hapfelmeier, S. , Freedrich, S. , Weber, T. C. , Kirundi, J. , Suar, M. , McCoy, K. D. , von Mering, C. , Macpherson, A. J. , & Hardt, W.‐D. (2010). Like will to like: Abundances of closely related species can predict susceptibility to intestinal colonization by pathogenic and commensal bacteria. PLoS Pathogens, 6, e1000711. 10.1371/journal.ppat.1000711 20062525 PMC2796170

[ece310964-bib-0045] Steinhauer, N. , Kulhanek, K. , Antúnez, K. , Human, H. , Chantawannakul, P. , Chauzat, M.‐P. , & vanEngelsdorp, D. (2018). Drivers of colony losses. Current Opinion in Insect Science, 26, 142–148. 10.1016/j.cois.2018.02.004 29764654

[ece310964-bib-0046] Stephan, J. G. , Lamei, S. , Pettis, J. S. , Riesbeck, K. , de Miranda, J. R. , & Forsgren, E. (2019). Honeybee‐specific lactic acid bacterium supplements have no effect on American foulbrood‐infected honeybee colonies. Applied and Environmental Microbiology, 85, e00606‐19. 10.1128/AEM.00606-19 31003985 PMC6581185

[ece310964-bib-0047] Truong, A.‐T. , Kang, J. E. , Yoo, M.‐S. , Nguyen, T. T. , Youn, S.‐Y. , Yoon, S.‐S. , & Cho, Y. S. (2023). Probiotic candidates for controlling *Paenibacillus larvae*, a causative agent of American foulbrood disease in honey bee. BMC Microbiology, 23, 150. 10.1186/s12866-023-02902-0 37226109 PMC10207761

[ece310964-bib-0048] Vásquez, A. , Forsgren, E. , Fries, I. , Paxton, R. J. , Flaberg, E. , Szekely, L. , & Olofsson, T. C. (2012). Symbionts as major modulators of insect health: Lactic acid bacteria and honeybees. PLoS One, 7, e33188. 10.1371/journal.pone.0033188 22427985 PMC3299755

[ece310964-bib-0049] Vásquez, A. , Olofsson, T. C. , & Sammataro, D. (2009). A scientific note on the lactic acid bacterial flora in honeybees in the USA – A comparison with bees from Sweden. Apidologie, 40, 26–28. 10.1051/apido:2008063

[ece310964-bib-0050] Ye, M.‐H. , Fan, S.‐H. , Li, X.‐Y. , Tarequl, I. M. , Yan, C.‐X. , Wei, W.‐H. , Yang, S.‐M. , & Zhou, B. (2021). Microbiota dysbiosis in honeybee (*Apis mellifera* L.) larvae infected with brood diseases and foraging bees exposed to agrochemicals. Royal Society Open Science, 8, 201805. 10.1098/rsos.201805 33614099 PMC7890499

[ece310964-bib-0051] Zendo, T. , Ohashi, C. , Maeno, S. , Piao, X. , Salminen, S. , Sonomoto, K. , & Endo, A. (2020). Kunkecin A, a new nisin variant bacteriocin produced by the fructophilic lactic acid bacterium, *Apilactobacillus kunkeei* FF30‐6 isolated from honey bees. Frontiers in Microbiology, 11, 571903. 10.3389/fmicb.2020.571903 33042078 PMC7525160

